# Spermine and Citrate as Metabolic Biomarkers for Assessing Prostate Cancer Aggressiveness

**DOI:** 10.1371/journal.pone.0062375

**Published:** 2013-04-23

**Authors:** Guro F. Giskeødegård, Helena Bertilsson, Kirsten M. Selnæs, Alan J. Wright, Tone F. Bathen, Trond Viset, Jostein Halgunset, Anders Angelsen, Ingrid S. Gribbestad, May-Britt Tessem

**Affiliations:** 1 MI Lab, Department of Circulation and Medical Imaging, Norwegian University of Science and Technology (NTNU), Trondheim, Norway; 2 St. Olavs Hospital, Trondheim University Hospital, Trondheim, Norway; 3 Department of Laboratory Medicine and Children’s and Women’s Health, NTNU, Trondheim, Norway; 4 Department of Urology, St. Olavs Hospital, Trondheim University Hospital, Trondheim, Norway; 5 Department of Radiology, Radboud University Nijmegen Medical Center, Nijmegen, The Netherlands; 6 Department of Pathology and Medical Genetics, St. Olavs Hospital, Trondheim University Hospital, Trondheim, Norway; 7 Department of Cancer Research and Clinical Medicine, NTNU, Trondheim, Norway; Instituto de Investigación Sanitaria INCLIVA, Spain

## Abstract

Separating indolent from aggressive prostate cancer is an important clinical challenge for identifying patients eligible for active surveillance, thereby reducing the risk of overtreatment. The purpose of this study was to assess prostate cancer aggressiveness by metabolic profiling of prostatectomy tissue and to identify specific metabolites as biomarkers for aggressiveness. Prostate tissue samples (n = 158, 48 patients) with a high cancer content (mean: 61.8%) were obtained using a new harvesting method, and metabolic profiles of samples representing different Gleason scores (GS) were acquired by high resolution magic angle spinning magnetic resonance spectroscopy (HR-MAS). Multivariate analysis (PLS, PLS-DA) and absolute quantification (LCModel) were used to examine the ability to predict cancer aggressiveness by comparing low grade (GS = 6, n = 30) and high grade (GS≥7, n = 81) cancer with normal adjacent tissue (n = 47). High grade cancer tissue was distinguished from low grade cancer tissue by decreased concentrations of spermine (p = 0.0044) and citrate (p = 7.73·10^−4^), and an increase in the clinically applied (total choline+creatine+polyamines)/citrate (CCP/C) ratio (p = 2.17·10^−4^). The metabolic profiles were significantly correlated to the GS obtained from each tissue sample (r = 0.71), and cancer tissue could be distinguished from normal tissue with sensitivity 86.9% and specificity 85.2%. Overall, our findings show that metabolic profiling can separate aggressive from indolent prostate cancer. This holds promise for the benefit of applying *in vivo* magnetic resonance spectroscopy *(*MRS) within clinical MR imaging investigations, and HR-MAS analysis of transrectal ultrasound-guided biopsies has a potential as an additional diagnostic tool.

## Introduction

Currently there are no objective clinical tools that can accurately discriminate aggressive from indolent prostate cancer. The Gleason scoring system [1] is the most important prognostic tool in treatment planning, but it is dependent on subjective factors in the evaluation of aggressiveness and is limited by underestimation due to under-sampling of biopsies. New diagnostic and prognostic tools for evaluating prostate cancer aggressiveness are therefore urgently needed. Metabolic alteration is an emerging hallmark of cancer [2], and metabolic profiling of prostate tissue using magnetic resonance spectroscopy (MRS) can provide additional information about tumor behaviour [3], especially with the possibility to translate findings from *ex vivo* tissue samples to *in vivo* measurements in patients using MRS imaging (MRSI).

Metabolic differences between prostate cancer and normal tissue are documented both *in vivo* by MRSI [4], [5], [6], [7] and *ex vivo* using high resolution magic angle spinning MRS (HR-MAS) [8], [9], [10]. In some hospitals, MRSI has already been implemented into clinical practice, making use of the (total choline+creatine+polyamines)/citrate (CCP/C) ratio or the (total choline+creatine)/citrate (CC/C) ratio which is increased in malignant prostate tissue [5], [8], [11], [12]. The total choline signal measured *in vivo* can be separated by HR-MAS into the choline-containing metabolites [free choline (Cho), phosphocholine (PCho) and glycerophosphocholine (GPC)] [8], [9], [13]. Lactate and alanine are also reported to be increased in cancer compared to normal tissues [10], while the prostate-specific metabolites citrate and the polyamines (spermine, spermidine, and putrescine) are found in lower concentrations in cancer tissue [9], [14].

HR-MAS is a well-established technique for analyzing biological tissue, leaving the samples unprocessed for subsequent histopathological evaluation or other molecular methods such as gene expression profiling [15], [16]. We have previously confirmed that there is a significant correlation between results from *ex vivo* HR-MAS analyses and *in vivo* MRSI from spatially matched regions, proving that the translation from *ex vivo* to *in vivo* is valid [17]. The overall aim of this study was to investigate the possibility of assessing prostate cancer aggressiveness by HR-MAS analysis of human prostate tissue, and to identify specific metabolites as biomarkers for cancer aggressiveness. The study was performed using fresh frozen tissue samples extracted from radical prostatectomy specimens using a novel method allowing samples with a high cancer content to be included [18]. Both metabolic profiles and individual metabolite concentrations were used to discriminate between the histologically determined Gleason score (GS) which was evaluated from a cryosection of each tissue sample. The value of HR-MAS as an additional tool to complement histopathological scoring, and the improvement the results add to *in vivo* MRSI examinations, will be discussed.

## Materials and Methods

### Patient and Tumor Characteristics

Since 2007, all prostate cancer patients at St. Olavs Hospital, Trondheim University Hospital, Norway, scheduled for radical prostatectomy have been invited to sign an informed consent form to donate tissue for research. From each patient a 2 mm transversal prostate tissue slice has been collected for storage in the Regional Research Biobank of Central Norway. The study has been approved by the Regional Committees for Medical and Health Research Ethics (REC) Central, Norway, and the Data Inspectorate of Norway. The current study includes 48 patients with no previous prostate cancer treatment and with a tumor volume >5% of the gland, estimated by histopathology. Patient characteristics are described in Table 1.

**Table 1:** Characteristics of patients and prostate tissue samples.

**Age (mean, range)**	Years	62.0(48–69)
**Tumor volume (mean, range)**	Percentage of prostate gland	21.4 (5–90)
**sPSA (mean, range)**	Before surgery (ng/mL)	10.5 (3.7–45.8)
	After surgery (ng/mL)*	0.0 (0.0–1.0)
**pT stage (patients)**	pT2a	2
	pT2b	1
	pT2c	29
	pT3a	7
	pT3b	7
	unknown	2
**Gleason score of HR MAS tissue samples (samples/patients)^a^**	0	47/41
	3+3	30/21
	3+4	22/19
	4+3	20/15
	4+4	16/12
	3+5	2/1
	5+3	1/1
	4+5	12/9
	5+4	8/5

* 3 months after prostatectomy^a^ Several samples from each slice (range: 1–7 samples per slice depending on tumor size) were selected from locations corresponding to cancer and normal areas, resulting in a total of 158 HR-MAS samples representing the different Gleason grades.

### Harvesting Method and Sselection of HR-MAS Samples

**Figure 1:**
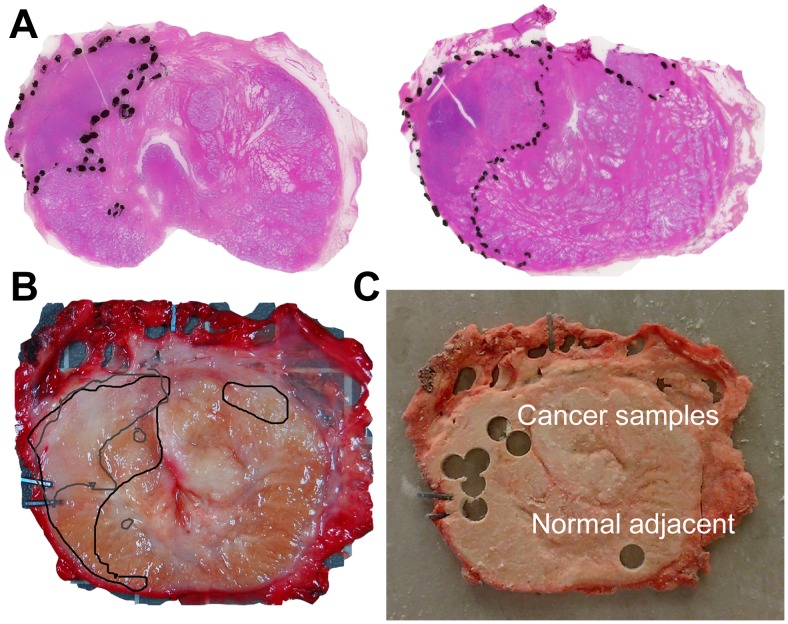
The prostate sample harvesting method after radical prostatectomy. (A) The two HES-stained sections adjacent to the tissue slice. (B) To localize the cancer and normal areas, micrographs of the two HES stained histological sections adjacent to the removed tissue slice were fused with a photograph of the frozen tissue slice. The regions of interest were marked and transferred to a transparency sheet to be used as a map for guiding sample extraction. (C) Cylindrical samples (3 mm diameter) for HR-MAS were excised from regions with normal tissue and cancer tissue with different Gleason grades. The Gleason grade and the percentages of benign glandular tissue, stroma and cancer tissue were verified by analyzing a 4 µm cryosection from each extracted sample. The figure is adapted from reference [36].

On average 15 minutes after surgical removal of the prostate gland, a tissue slice (2 mm) was obtained by transection through its middle, perpendicularly to the urethra [18]. The slice was snap frozen by clamping between two metal plates precooled in liquid nitrogen and stored at −80°C. The two remaining halves were stitched to a cork board, in order to avoid disturbances in the histopathological evaluation of the surgical margin. After fixation in formalin, both halves were further sliced (4 mm thick slices) and paraffin embedded. Microscopic sections were made and stained with hematoxylin, erythrosine and saffron (HES) for diagnostic purposes. The HR-MAS samples were excised from the frozen prostate slice using a novel harvesting method described by Bertilsson et al. [18]. By using this method, summarized in Figure 1, tissue samples of predetermined histopathological GS are obtained from the slice. During sample extraction, the frozen tissue slice was placed on an aluminium plate in direct contact with liquid nitrogen, preventing the tissue from thawing and thus reducing molecular degradation. Several samples from each slice (range: 1–7 samples per slice (median: 3) depending on tumor size) were selected from malignant areas of different GS and from normal adjacent areas, using the HES stained slides from neighboring tissue blocks as a guide. Thus, a total of 162 HR-MAS samples was obtained. Normal adjacent samples are defined as samples not showing signs of cancer, thus containing only benign glandular and/or stromal tissue, and these samples were excised as far away from the cancer as possible. To assess the GS of each HR-MAS sample (2 mm thick), and to determine the amount of cancer tissue, stroma, and glandular tissue, a 4 µm cryosection was cut from one side of the extracted sample and HES stained, and the tissue composition was evaluated by an experienced pathologist specialized in uropathology before the HR-MAS procedure. The samples were not thawed before the moment they go into the magnet, reducing additional freeze-thaw effects. There are no studies stating that long-term storage at −80°C (up to 5 years) affect metabolism.

### HR-MAS MRS Experiments

A PBS solution (3 µl) containing trimethylsilyl 3-propionic acid sodium salt (TSP, 5 mM) and formate (25 mM) was added to disposable Kel-F HR-MAS inserts (30 µl, Bruker Biospin, Germany). Each prostate tissue sample (mean weight: 12.7 mg, range: 3.0–21.9 mg) was transferred to a HR-MAS insert using a sterile biopsy punch (2 mm, Miltex Gmbh, Germany), and the insert was placed into the zirconium rotor (4 mm). HR-MAS was performed on a Bruker Avance DRX600 (14.1 T) spectrometer (Bruker BioSpin, Germany) equipped with a ^1^H/^13^C MAS probe. Proton spectra were acquired at 4°C with a spin rate of 5 kHz. Pulse-acquired spectra were obtained with a presaturation delay of 3.0s and acquisition time of 3.27s. A Carr-Purcell-Meiboom-Gill (CPMG) spin echo sequence [90°-(τ-180°-τ)_n_ –acquisition] was used to suppress signals from lipids and macromolecules with an effective echo time of 60 ms. One hundred and twenty-eight scans over a spectral region of 10 kHz were collected into 64k points for both sequences. The spectra were Fourier transformed with a line broadening of 0.30 Hz. Chemical shifts were referenced to the lactate peak (left peak of the doublet) at 1.336 ppm and a linear baseline correction was applied (Topspin 3.1, Bruker Biospin, Germany). Peak assignments were set according to the human metabolomics database and previous published papers using HR MAS on prostate tissue [9], [10], [19].

### Multivariate Analysis

The spectral data between 1.46 and 4.66 ppm from the CPMG spectra were used for multivariate analysis. The spectra were normalized to an equal total area and peak aligned using icoshift [20]. Signals from ethanol contamination (3.65–3.69 ppm) were removed from the spectra together with those of lipid residuals at 1.60, 2.05, and 2.27 ppm. Preprocessing of the spectra was performed in MATLAB 7.8.0 (The Mathworks, Inc., USA). In addition to principal component analysis (PCA), partial least squares (PLS) regression and PLS discriminant analysis (PLS-DA) [21] were used to model the relationship between the MR spectra and tumor/patient characteristics (tissue composition, GS, serum PSA (sPSA), tumor volume, age and pT-stage). In order to avoid overfitting, double cross-validation was performed [22]. A PLS model was built on training samples (80% of the data set) and used to predict the status of independent test samples (the remaining 20%). The optimal number of LVs (latent variables) to use in the model was determined by cross-validation of the training data and applied independently to the test data. Both the inner and outer loops of the double cross-validation procedure were repeated 20 times with different randomly chosen training and test sets, and the average results are presented. As several samples from each patient were analyzed, spectra from one patient were put in either the training or the test set. The variable importance was evaluated by variable importance in projection (VIP) scores [23]. Variables with a VIP score greater than one are generally considered to be important The classification results were validated by permutation testing (n = 1000, significance for p<0.05) [22]. Multivariate analyses were performed in MATLAB using PLS_toolbox 6.2.1 (Eigenvector Research, Inc., USA).

### Absolute Quantification of Metabolites by LCModel

The pulse-acquired spectra were quantified using LCModel [24], [25] based on a novel basis set of 23 metabolites. The basis set of simulated metabolite spectra was generated using NMRSIM (Bruker BioSpin, Germany), and the metabolites were quantified between 4.72 ppm and −0.8 ppm. The baseline was modeled with a cubic spline function with a maximum of two knots, and macromolecules were included in the fitting, simulated with single peaks including prior knowledge of line width, chemical shift, and relative amplitude. Small molecule metabolite and lipid chemical shifts were set as mean values based on an initial assignment of spectra from 10 samples of varying tissue type. For metabolites where some peaks were not clearly resolved in these spectra (GPC, GPE, glucose, and the amino acids), literature values were used [26], [27], [28]. Ethanol, a contaminant in some samples, was included in the basis set for a successful subsequent fitting with the metabolite spectra. The metabolites were quantified according to formate and the concentrations are reported as mmol/kg wet weight. Full relaxation of formate was assured by using results from T1 relaxation measurements performed on six additional tissue samples.

### Statistical Analysis of Metabolite Concentrations

Differences in metabolite concentrations between cancer and normal adjacent tissue, and metabolic differences related to aggressiveness (low grade (GS = 6) vs. high grade (GS≥7)) were analyzed by linear mixed models, accounting for the effect of samples originating from the same patient. Individual comparison of samples of GS 6, 7, and 8–9, in addition to differences between samples of GS 3+4 and 4+3 were also tested. Analyses were performed in R (version 2.14.1, R Foundation for Statistical Computing) with the lme4 package [29]. The data were log transformed prior to analysis in order to obtain normally distributed residuals. The Benjamini and Hochberg false discovery rate was used to correct for multiple testing. Adjusted p-values<0.05 were considered significant.

## Results

### Samples

**Figure 2:**
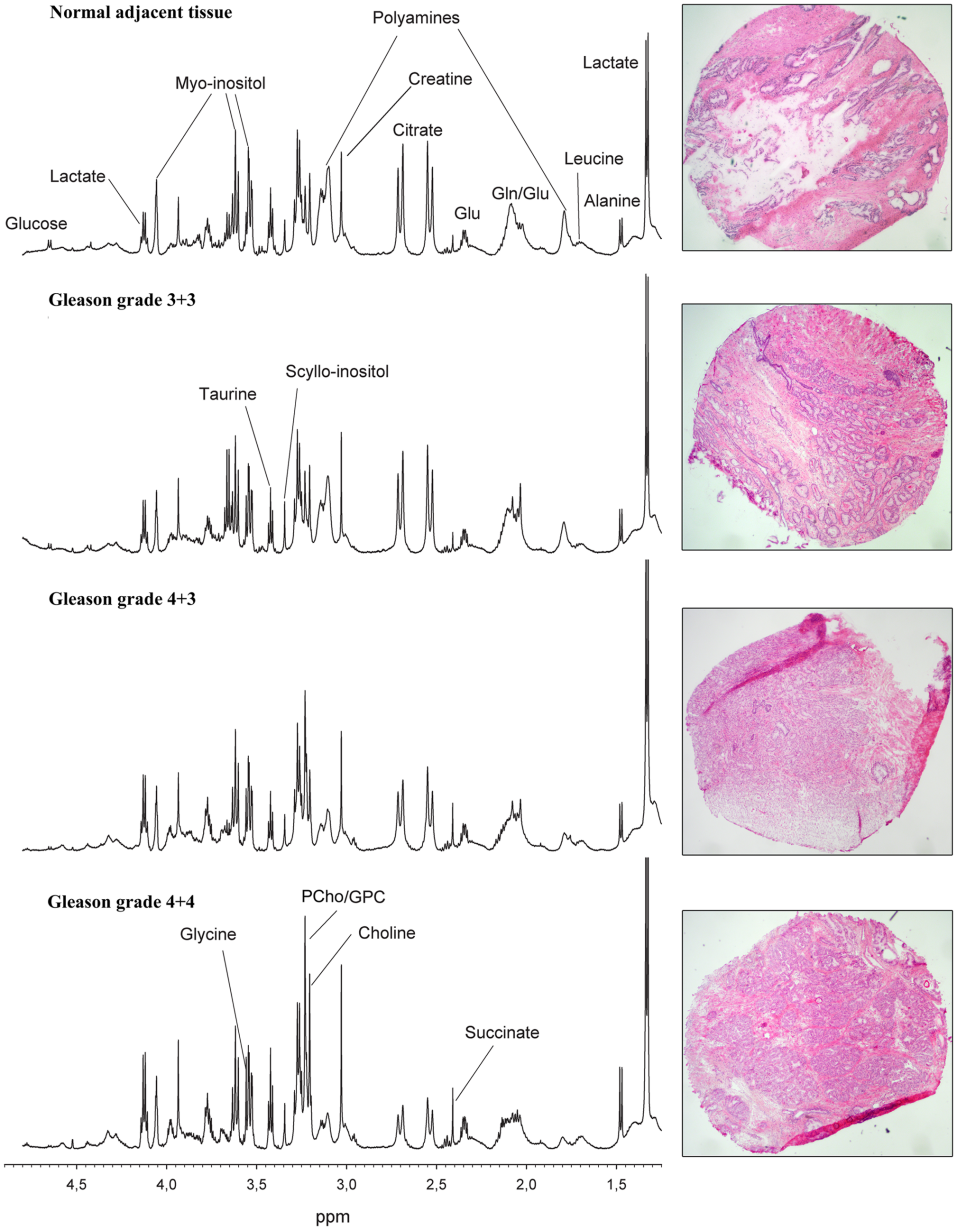
Representative HR-MAS spectra and corresponding HES stained prostate tissue samples with different Gleason grades. Visual inspection of the spectra show decreased levels of polyamines (predominately spermine) and citrate, and increased levels of GPC, PCho, and Cho with increasing tumor grade.

The PCA score plot of the CPMG spectra (n = 162) revealed four outlying samples. These samples were removed from the data set due to very high lipid concentrations and microscopic evidence of severe inflammation. Of the 158 samples included in this study, 47 were shown to contain only normal prostate tissue components, while 111 samples contained cancer tissue. The average cancer content was 61.8% (range: 10–100%) and 30 cancer samples were defined as low grade (GS 6) while 81 samples were defined as high grade (GS 7–9). Sample and patient characteristics are summarized in Table 1. Representative HR-MAS spectra and the corresponding histopathological image of normal prostate tissue and cancer tissue with different Gleason grades are shown in Figure 2.

### Metabolic Profiles Related to Clinical Parameters

The metabolic profiles were correlated to tissue composition (percentage of benign glandular tissue: r = 0.67, stroma: r = 0.70, and cancer: r = 0.77) (p<0.001). The metabolic profiles were not significantly correlated to the patient’s sPSA level, tumor volume, age or pT-stage (p>0.05).

### Distinguishing Cancer and Normal Adjacent Tissue

#### Multivariate analysis

**Figure 3:**
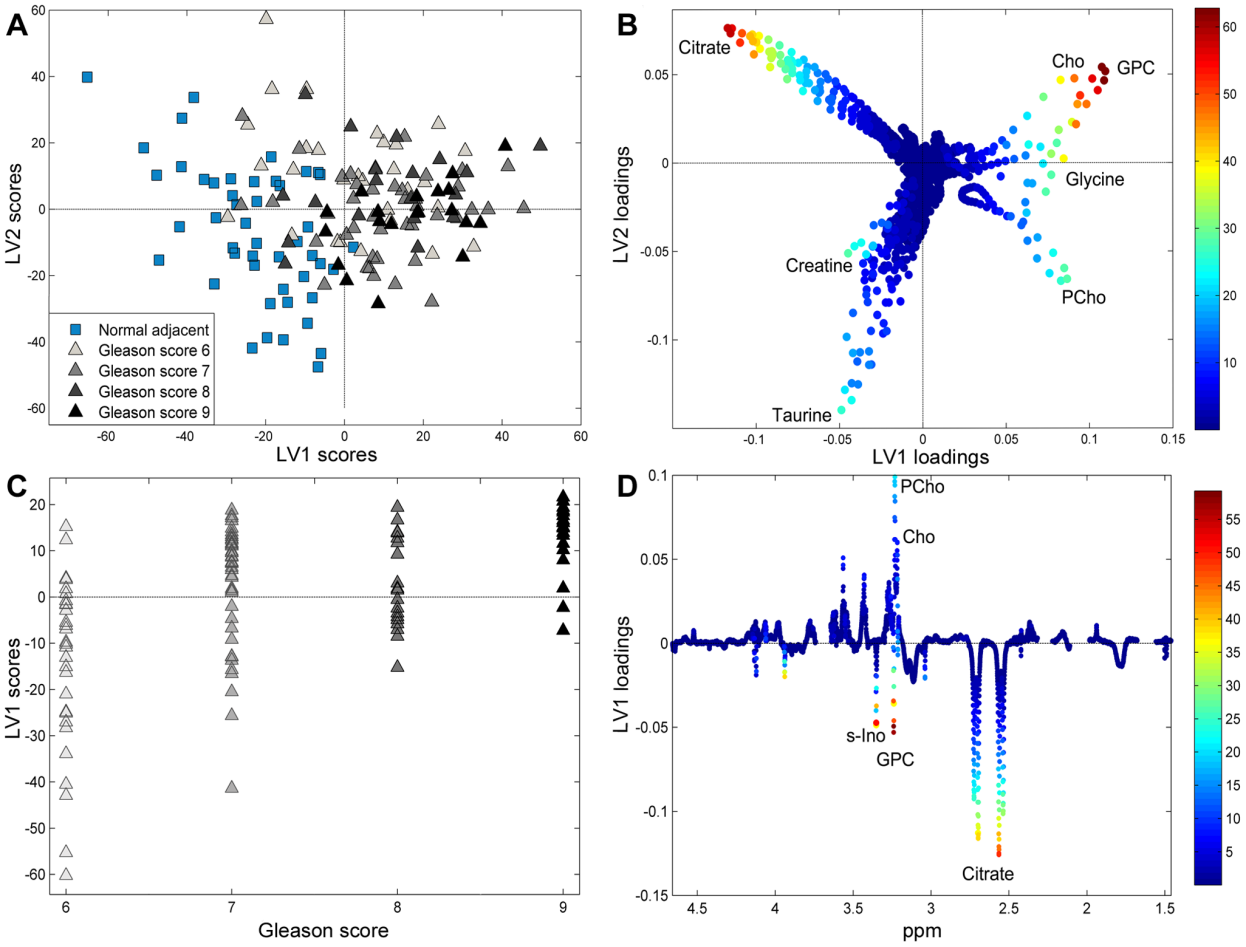
Prostate cancer metabolic profiles are correlated to aggressiveness. (A) PLS scores and (B) loadings of LV1 and LV2 from PLS regression correlating the metabolic profiles to GS with a correlation coefficient r = 0.71. The cancer samples are separated from the normal samples in the score plot, with the loadings showing metabolic alterations related to malignancy. Samples with GS 9 are almost completely separated from normal adjacent samples in the score plot, while some samples with a lower score overlap with the normal ones. The PLSDA model explains 48.2% of the x-variance and 53.7% of the y-variance (C) PLS scores and (D) the corresponding loading profile of LV1 from PLS regression of the cancer samples only, correlating the metabolic profiles to GS with a correlation coefficient r = 0.45. The resulting model explains 20.0% of the x-variance and 27.4% of the y-variance of the data. The loadings in (B) and (D) are colored according to their VIP score. S-ino; scyllo-inositol.

Based on the metabolic profiles, cancer and normal samples were separated with 86% correct classification using PLS-DA on independent test samples (sensitivity 86.9%, specificity 85.2%, p<0.001). A PLS model correlating the metabolic profiles to GS (Figure 3, A-B) separates the normal adjacent tissue samples from the cancer tissue samples. The loadings showed decreased levels of citrate, taurine and creatine, and an increase in GPC, PCho, Cho, and glycine in cancer compared to normal tissue.

#### Absolute quantification by LCModel

The quantified metabolite concentrations in cancer and normal tissue samples (n = 153) are shown in Table 2. Five spectra were not quantified due to insufficient fitting caused by high lipid signals.

**Table 2:** Metabolite concentrations (mmol/kg) in cancer and normal prostate tissue samples.

Metabolite	Normal adjacent samples	Cancer samples	p-value^b^
	(n = 47)	(n = 106)	
	Median (IQR)	Median (IQR)	
Spermine^a^	1.92 (0.86–3.13)	1.22 (0.66–2.00)	0.022*
Putrescine	0.38 (0.00–0.97)	0.02 (0.00–0.25)	2.07·10^−4^*
Cho^a^	0.46 (0.32–0.64)	1.02 (0.65–1.59)	6.89·10^−9^*
PCho^a^	0.34 (0.19–0.51)	0.70 (0.39–1.12)	5.68·10^−6^*
GPC^a^	0.42 (0.25–0.51)	0.78 (0.48–1.17)	2.04·10^−6^*
GPE	0.22 (0.00–0.42)	0 (0.00–0.51)	0.387
PE^a^	1.66 (1.10–2.39)	2.67 (1.90–3.69)	1.38·10^−5^*
Eth	0.00 (0.00–0.06)	0.00 (0.00–0.21)	0.926
Lactate^a^	12.34 (9.79–16.71)	18.20 (13.90–24.45)	7.52·10^−5^*
Alanine^a^	1.71 (1.22–2.09)	2.15 (1.65–2.79)	0.0014*
Glucose	0.90 (0.53–1.36)	0.00 (0.00–0.42)	5.70·10^−12^*
Citrate^a^	9.87 (5.14–14.32)	6.41 (3.34–9.46)	0.049*
Succinate^a^	0.38 (0.30–0.49)	0.59 (0.46–0.81)	1.20·10^−4^*
Creatine^a^	2.43 (1.76–3.11)	2.09 (1.64–2.58)	0.820
Glutamate^a^	2.69 (2.28–3.56)	4.82 (3.61–6.88)	2.60·10^−9^*
Glutamine^a^	1.98 (1.56–2.37)	2.74 (2.25–3.52)	1.78·10^−5^*
Glycine^a^	1.53 (1.18–1.98)	2.50 (1.74–3.18)	2.04·10^−6^*
Isoleucine	0.09 (0.02–0.12)	0.17 (0.08–0.27)	0.0017*
Leucine	0.24 (0.17–0.34)	0.46 (0.30–0.64)	2.04·10^−6^*
Valine	0.21 (0.18–0.29)	0.38 (0.25–0.49)	7.66·10^−4^*
Taurine^a^	5.70 (3.88–6.32)	4.34 (3.65–6.53)	0.918
Myo-inositol^a^	8.82 (7.91–10.77)	9.22 (7.04–11.30)	0.435
Scyllo-inositol^a^	0.36 (0.25–0.58)	0.43 (0.33–0.59)	0.459

Concentrations are reported as mmol/kg wet weight. * p<0.05.

a Cramér Rao lower bound (CRLB, LCmodel uncertainty measure) lower than 20% of the concentration for more than 90% of the samples, which is acceptable for quantification [37], [38]. Higher CRLB values are the result of near or actual absence of signals in some samples.

b P-values from Linear mixed models corrected for multiple testing by Benjamini-Hochberg correction.

### Distinguishing Low Grade (GS = 6) and High Grade Cancer Tissue (GS≥7); Correlation with the Gleason System

#### Multivariate analysis

Metabolic profiles were correlated to GS with a correlation coefficient of r = 0.71 using PLS regression analysis (p<0.001) (Figure 3, A-B). When analyzing only the cancer samples, the metabolic profiles were correlated to GS with a correlation coefficient of r = 0.45 (p<0.001) (Figure 3, C-D). When dividing the samples into normal, high grade (GS≥7) and low grade (GS = 6), correct classification by PLS-DA was 85.8% (sensitivity 89.3%, specificity 82.3%), 77.4% (sensitivity 84.4%, specificity 70.5%), and 65.8% (sensitivity 64.1%, specificity 67.6%), respectively.

#### Absolute quantification by LCModel

The concentrations of spermine and citrate were shown to be significantly different between low grade and high grade cancers, while no significant differences were detected for the other metabolites. The concentrations and statistical results for the significant metabolites are summarized in Table 3. For further examination of the metabolite concentrations related to aggressiveness, metabolic differences between samples of GS 6, 7, and 8–9 were analyzed individually (Table 3). No significant differences between GS 7 and GS 8–9 were detected for any of the metabolites. In addition, no significant differences in metabolite concentrations were found between samples of GS 3+4 and 4+3 (p>0.05). The correlations between GS and the concentrations of spermine and citrate were r = −0.36 and r = −0.43, respectively.

**Table 3:** Metabolite concentrations (mmol/kg) and ratios in low grade (GS = 6) and high grade (GS≥7) prostate cancer samples and comparison between different GSs.

Metabolite/ratio	Low grade (n = 29)	High grade (n = 77)	p-value^a^	GS	GS	GS
				6 vs 7	6 vs 8–9	7 vs 8–9
	Median (IQR)	Median (IQR)		(p-value^a^)	(p-value^a^)	(p-value^a^)
**Spermine**	1.96 (1.23–3.72)	1.05 (0.54–1.57)	0.0044*	0.110	0.022*	0.769
**Citrate**	8.45 (7.20–14.82)	4.76 (2.95–7.78)	7.73·10^−4^*	0.014*	0.005*	0.769
**CCP/C**	0.78 (0.62–0.95)	1.20 (0.80–2.16)	2.17·10^−4^*	0.0016*	9.47·10^−4^*	0.162
**GPC/PCho**	1.53 (1.01–2.15)	1.02 (0.64–1.78)	0.0832	0.082	0.089	0.734

Concentrations are reported as mmol/kg wet weight. ^a^ P-values from Linear mixed models corrected for multiple testing by Benjamini-Hochberg correction; * p<0.05.

The clinically relevant CCP/C ratio was significantly increased in high grade compared to low grade cancer samples (Table 3). In addition, a trend of different GPC/PCho ratios between low and high grade cancer samples was detected (p = 0.08). When examining metabolite concentrations related to aggressiveness, the percentages of benign glandular, stroma, and cancer tissue were included in the linear mixed models in order to correct for differences in tissue composition. However, none of the tissue types had a significant contribution to the statistical models (p>0.05), and the results are presented without correction for tissue composition.

## Discussion

In this study performed using prostate tissue with high cancer content, we have shown the possibility to separate low grade from high grade prostate cancer using metabolic profiling. Decreased concentrations of citrate and spermine were shown to be valid MR tissue biomarkers for prostate cancer aggressiveness, and the metabolic profiles were significantly correlated to the GS showing that aggressive cancers have an altered metabolism compared to indolent cancer. Surprisingly, the choline containing components were not increasing with GS, indicating that spermine and citrate are the main contributors to the clinically applied CCP/C ratio which increases with GS. In addition, this study confirms the separation between cancer and normal tissue, and the HR-MAS metabolic profiles were successfully separated with 86.0% correct classification.

Many prostate cancer patients diagnosed with indolent disease (GS 6) are eligible for inclusion in active surveillance programs. It is therefore desirable to separate this group from patients with higher grade cancers. Citrate concentrations could separate samples with GS 6 from both GS 7 and 8–9, while the difference in spermine concentrations was only significant between GS 6 and GS 8–9. Interestingly, none of the metabolites was significantly different between samples with GS 7 and GS 8–9, indicating that samples with GS 7 (intermediate risk patients) have a metabolic pattern similar to higher grade cancers. This finding supports the consensus that only patients with GS≤6 should be included in active surveillance programs. Patients with GS 4+3 have worse prognosis than those with GS 3+4, however this study could not separate these clinically relevant subgroups.

Normal prostate epithelial cells produce and accumulate a large amount of citrate which is secreted as a major component of the prostatic fluid. Compared to normal tissue, decreased levels of citrate are previously observed in prostate cancer tissue by *ex vivo* MRS [9]. Our study confirms and extends these findings by demonstrating a significant negative correlation with GS, and significant differences between low grade and high grade cancer tissue, between samples of GS 6 and GS 7, and between GS 6 and GS 8–9. This supports the highly clinically relevant hypothesis that the citrate concentration can distinguish between aggressive and indolent prostate cancer.

Our results confirm previous *in vivo* and *ex vivo* MRS studies showing that a decrease in polyamines is associated with prostate cancer [8], [14], [30], [31]. Additionally, the very low putrescine concentration in our study confirms that the polyamine peak predominantly consists of spermine. Due to the significantly lower concentration of spermine in high grade compared to low grade tissue, we propose spermine as a discriminative MR biomarker for prostate cancer aggressiveness, and a focus to this should be considered using the CCP/C ratio in MRSI examinations. Today, spermine cannot be fully separated from the choline peak using MRSI, but due to rapid technological developments already in progress and higher field strengths (7T) making separation possible [32], polyamines and especially spermine are potential biomarkers in clinical practice.

Surprisingly, there were no significant differences between high grade and low grade prostate cancer in any of the quantified choline- or ethanolamine-containing metabolites (Eth, PE and GPE). Previous *ex vivo* studies have demonstrated significant correlations between GS and choline and total choline [33], and significantly higher concentrations of GPC in high grade (GS≥4+3) compared to low grade (GS≤3+4) cancers [13], which is not in accordance with our findings. We found a trend towards significance for the GPC/PCho ratio (p = 0.0832), which indicates a change in the choline-containing metabolites associated with increased aggressiveness, however not detected when examining the metabolites individually. Due to contradictory findings of choline metabolism also in other types of cancers [34], the choline metabolism related to cancer aggressiveness evidently needs further evaluation.

Previous *in vivo* MRSI studies have concluded a trend towards a correlation between the CCP/C ratio and prostate cancer aggressiveness [12], [35], and our study showed a highly significant difference in the CCP/C ratio between low and high grade cancers. Our findings on the individual metabolites, however, indicate that the decreased CCP/C ratio observed *in vivo* is mainly resulting from decreased citrate levels.

Although there was a correlation between the metabolic profiles and tissue composition, correction for tissue composition in the analysis of individual metabolite concentrations was not significant. This indicates that the metabolic differences between high and low grade prostate cancer samples are present independently of tissue composition. It is however likely that samples with lower cancer content would require statistical methods correcting for tissue composition.

A strength of this study is the inclusion of patients from the whole range of clinical stages, including patients with highly aggressive cancers. A limitation is however that the low grade tissue material (GS 6) was mainly acquired from patients having more aggressive tumors in the vicinity, and this may have induced metabolic perturbation in our low grade material. A sample cohort including more samples from patients with pure low grade cancer may provide even clearer metabolic differences between low and high grade cancers.

### Conclusion

Based on metabolic profiling of human prostate cancer samples this study shows that low and high grade prostate cancer tissue can be distinguished by the concentrations of spermine, citrate and the CCP/C ratio. In the future, by analyzing larger patient cohorts, concentration cut-off values can be determined for spermine and citrate, and models based on the metabolic profiles can become tools for assessing prostate cancer aggressiveness. HR-MAS is feasible as a diagnostic supplementary tool for evaluating transrectal ultrasound guided biopsies, providing metabolic profiles that can predict tumor aggressiveness. Ultimately, the translation from *ex vivo* measurements in tissue samples to a true non-invasive *in vivo* examination, rendered possible by improvements in MR technology, will be the main future goal. Thus, our results demonstrate the value of MRS in clinical treatment planning and as a tool for follow-up of patients included in active surveillance programs.
